# *Anadenanthera colubrina* regulated LPS-induced inflammation by suppressing NF-κB and p38-MAPK signaling pathways

**DOI:** 10.1038/s41598-024-66590-0

**Published:** 2024-07-11

**Authors:** Carolina Medeiros de Almeida Maia, Priscilla Guimarães Silva Vasconcelos, Silvana Pasetto, Walton Colby Godwin, Joanda Paolla Raimundo e Silva, Josean Fechine Tavares, Vanessa Pardi, Edja Maria Melo de Brito Costa, Ramiro Mendonça Murata

**Affiliations:** 1 Department of Dentistry, Postgraduate Program in Dentistry, State University of Paraiba, Campina Grande, Paraiba, Brazil; 2 Postgraduate Program in Natural and Synthetic Bioactive Products, Federal University of Paraiba, João Pessoa, Paraiba, Brazil; 3 Department of Foundational Sciences, School of Dental Medicine, East Carolina University, Greenville, NC USA; 4 Department of Biology, East Carolina University, Greenville, NC USA

**Keywords:** Phytotherapy, Polyphenols, Immune response, Anti-inflammatory agents, Inflammatory, Cytokines, TLR4 receptor, Gene expression analysis, Reverse transcription polymerase chain reaction, Oral diseases, Drug discovery, Drug development, Cytokines, Inflammation

## Abstract

We aimed to determine the chemical profile and unveil *Anadenanthera colubrina* (Vell.) Brenan standardized extract effects on inflammatory cytokines expression and key proteins from immunoregulating signaling pathways on LPS-induced THP-1 monocyte. Using the RT-PCR and Luminex Assays, we planned to show the gene expression and the levels of IL-8, IL-1β, and IL-10 inflammatory cytokines. Key proteins of NF-κB and MAPK transduction signaling pathways (NF-κB, p-38, p-NF-κB, and p-p38) were detected by Simple Western. Using HPLC-ESI-MS^n^ (High-Performance Liquid-Chromatography) and HPLC-HRESIMS, we showed the profile of the extract that includes an opus of flavonoids, including the catechins, quercetin, kaempferol, and the proanthocyanidins. Cell viability was unaffected up to 250 µg/mL of the extract (LD_50_ = 978.7 µg/mL). Thereafter, the extract's impact on the cytokine became clear. Upon LPS stimuli, in the presence of the extract, gene expression of IL-1β and IL-10 were downregulated and the cytokines expression of IL-1β and IL-10 were down an upregulated respectively. The extract is involved in TLR-4-related NF-κB/MAPK pathways; it ignited phosphorylation of p38 and NF-κB, orchestrating a reduced signal intensity. Therefore, *Anadenanthera colubrina*'s showed low cytotoxicity and profound influence as a protector against the inflammation, modulating IL-1β and IL-10 inflammatory cytokines gene expression and secretion by regulating intracellular NF-κB and p38-MAPK signaling pathways.

## Introduction

Pathogen-Associated Molecular Patterns (PAMPs) are molecular structures that are commonly found on various pathogens such as bacteria, viruses, fungi, and parasites. These patterns are recognized by the innate immune system as potentially harmful, triggering a series of immune responses aimed at eliminating the invading pathogens^1^. In the oral cavity, various PAMPs are found in the oral microbiome, including fungal/bacterial cell wall components such as β-Glucans, Mannans and Chitin found in fungi like *Candida albicans*^2,3^. Another critical PAMPs in the oral microbiome are the bacterial cell wall components such as Peptidoglycans, Lipoteichoic Acids (LTA) and Lipopolysaccharides (LPS)^1,4^. The PAMPs recognition can activate strong immune responses, as well as trigger an associated inflammatory pattern of different oral conditions, such as periodontal diseases^1,4,5^.

Recognition of these PAMPs by pattern recognition receptors (PRRs) expressed on cells of the innate immune system, such as macrophages, dendritic cells, and epithelial cells, triggers signaling pathways that lead to the activation of immune responses. Recognition of oral microbiome PAMPs ligands occurs via some of the Toll-like receptors (TLRs), NOD receptors, RIG receptors, Dectin receptor and C-lectin receptors (CLRs) whereas a signaling cascade is initiated that culminates in proinflamatory cytokine and chemokine production ^1,2,6^. Thus, an associated inflammatory pattern of the condition is stablished ^5,7–9^.

The signaling sequence through NF‐κB and MAPK pathways may also be activated once the TLRs recognize other microorganisms’ PAMPs such as the LPS produced by *Porphyromonas gingivalis*, which is a major glycolipid within the outer membrane of gram-negative bacteria. *P. gingivalis* is highly associated with periodontitis pathogenesis, whereas LPS presence serves as a critical virulence factor triggering an onset inflammation pattern driven by the cytokines and inflammatory mediators release that exacerbate the immune response and local tissues damage^5,10–12^. Although the host immune response is an important factor against infections, when these inflammatory axes become dysregulated, they may also be associated with collateral damage to the host, exacerbation of infection effects, and it plays an important role in carcinogenesis^13^.

The development of effective anti-inflammatory drugs becomes essential in order to mitigate the risks associated with imbalances in immune response and inflammation^14,15^. Considering the pharmacological significance of plant-derived substances, significant attention has been drawn to them, especially to discovering potential therapeutic agents for treating a wide range of disorders^16–18^.

In this context, *Anadenanthera colubrina* (Vell.) Brenan, popularly known as “angico”, is widely applied in Brazilian folk medicine to treat local inflammation^19,20^. In our prior study, the *A. colubrina* standardized extract exhibited a significant reduction in *C. albicans* infection and effectively modulated the *Candida*-induced inflammatory response in human gingival fibroblasts^21^. However, it remains unknown whether the therapeutic effect is attributed to a specific active compound or a complex mixture of active substances within the extract—the phytocomplex. Despite the anti-inflammatory properties of *A. colubrina*, there is limited data on its immunomodulatory effects on transcriptome and proteome levels and molecular signaling transductions. Therefore, in the present study we aimed to investigate the phytochemical profile, the in vitro immunomodulatory effect of the standardized extract of *A. colubrina* bark, and the underlying cell signaling transduction pathways in LPS-stimulated monocytes.

## Results

### Cytotoxicity

**Figure 1:**
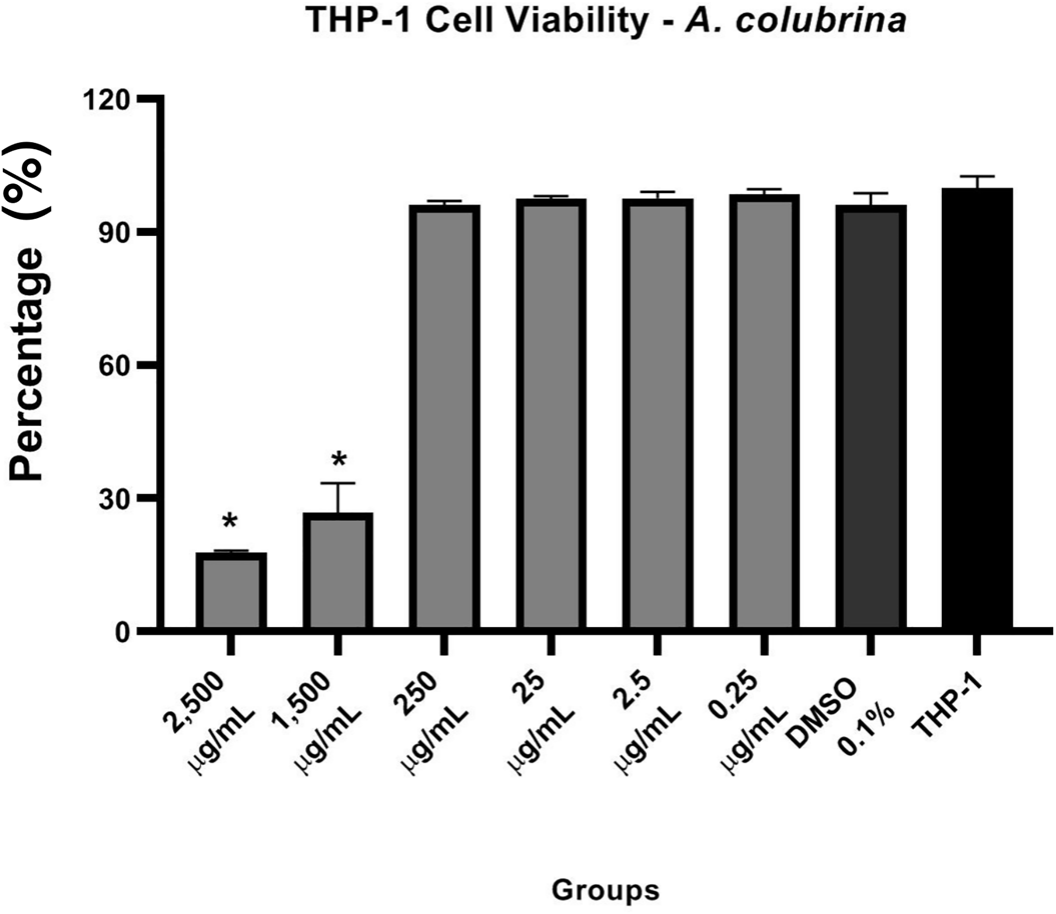
Cytotoxic effect of *A. colubrina* extract (2,500 µg/mL – 0.25 µg/mL) on THP-1 monocytes after 24 h of treatment (LD_50_ = 978.7 µg/mL). THP-1: only cells; DMSO 0.1%: vehicle control. Values shown with an asterisk (*) are statistically significant when compared to the vehicle control (*p* < 0.05).

Using THP-1 cells, we first asked if the *A. colubrina* extract could treat the cells without killing them. So, we evaluated its cytotoxicity, and the experiments presented LD_50_ of 978.7 µg/mL and a non-toxic profile on THP-1 cells culture, with no significant effect on cell viability in concentrations up to 250 µg/mL when compared to the vehicle and the cell control (Fig. 1).

### Modulatory effect on inflammatory cytokines gene expression

**Figure 2:**
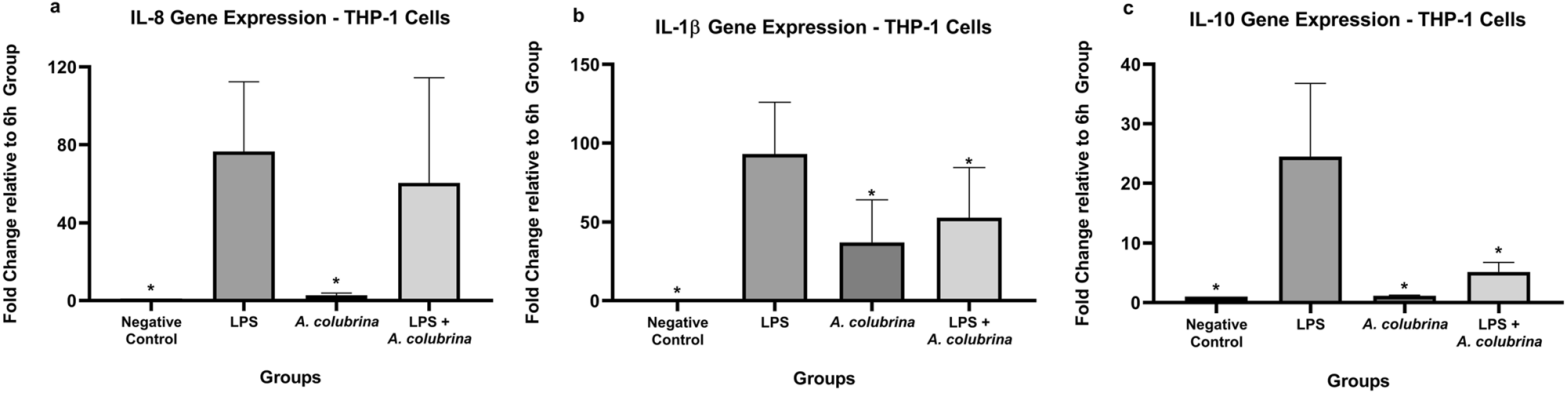
Real-time quantitative information about to relative gene expression of a) IL-8, b) IL-1β, and c) IL-10 of THP-1 cells after 6 h of treatment with *A. colubrina* extract (250 µg/mL) and stimulation by LPS (100 ng/mL). Values are shown as the fold-change relative to the negative control group (6 h). Values shown with an asterisk (*) are statistically significant when compared to the negative control (*p* < 0.05).

Following prior studies showing the extract presents anti-inflammatory effects, we decided to perform experiments for gene expression where the results showed the *A. colubrina* extract at 250 µg/mL presented modulatory effects on the gene expression of inflammatory cytokines. The extract itself up-regulated the gene expression of IL-1β cytokine. Upon LPS stimuli, gene expression of IL-1β and IL-10 were downregulated by *A. colubrina*, while it was ineffective in modulating IL-8 gene expression (Fig. 2a,b,c).

After that, to better understand the potential of the standardized extract, we also choose to measure the pro and anti-inflammatory cytokines secretion followed by signaling pathways regulation.

### Pro and anti-inflammatory cytokines secretion and signaling pathways regulation

**Figure 3:**
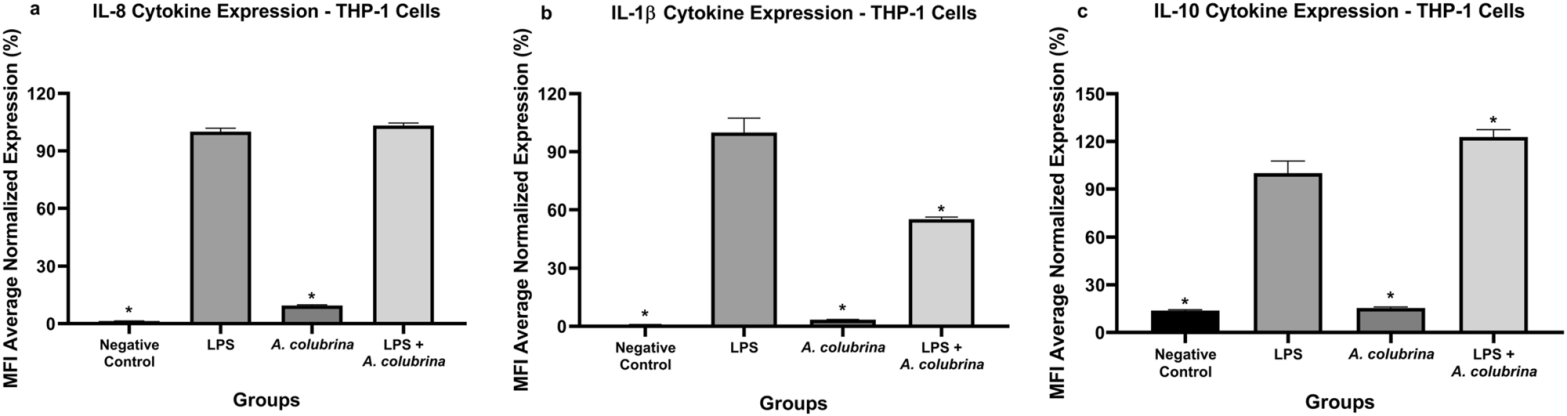
Pro and anti-inflammatory cytokines expression of a) IL-8, b) IL-1β, and c) IL-10 by THP-1 cells after 6 h of treatment with A. *colubrina* extract (250 µg/mL). Values shown with an asterisk (*) are statistically significant when compared to the LPS-induced cells group (*p* < 0.05).

The expression of pro-inflammatory (IL-8 and IL-1β) and anti-inflammatory (IL-10) cytokines after treatments with *A. colubrina* extract (250 µg/mL) were assessed using the THP-1 culture supernatants. The extract itself did not induce any inflammatory response. Also, it did not reduce IL-8 cytokine secretion from the LPS-induced THP-1 cells group (*p* < 0.05). However, in the LPS-stimulated group treated with *A. colubrina*, IL-1β, and IL-10 cytokines levels expression were significantly reduced and increased, respectively (*p* < 0.05), when compared to the LPS-induced only cells group (Fig. 3a, b, c).

**Figure 4:**
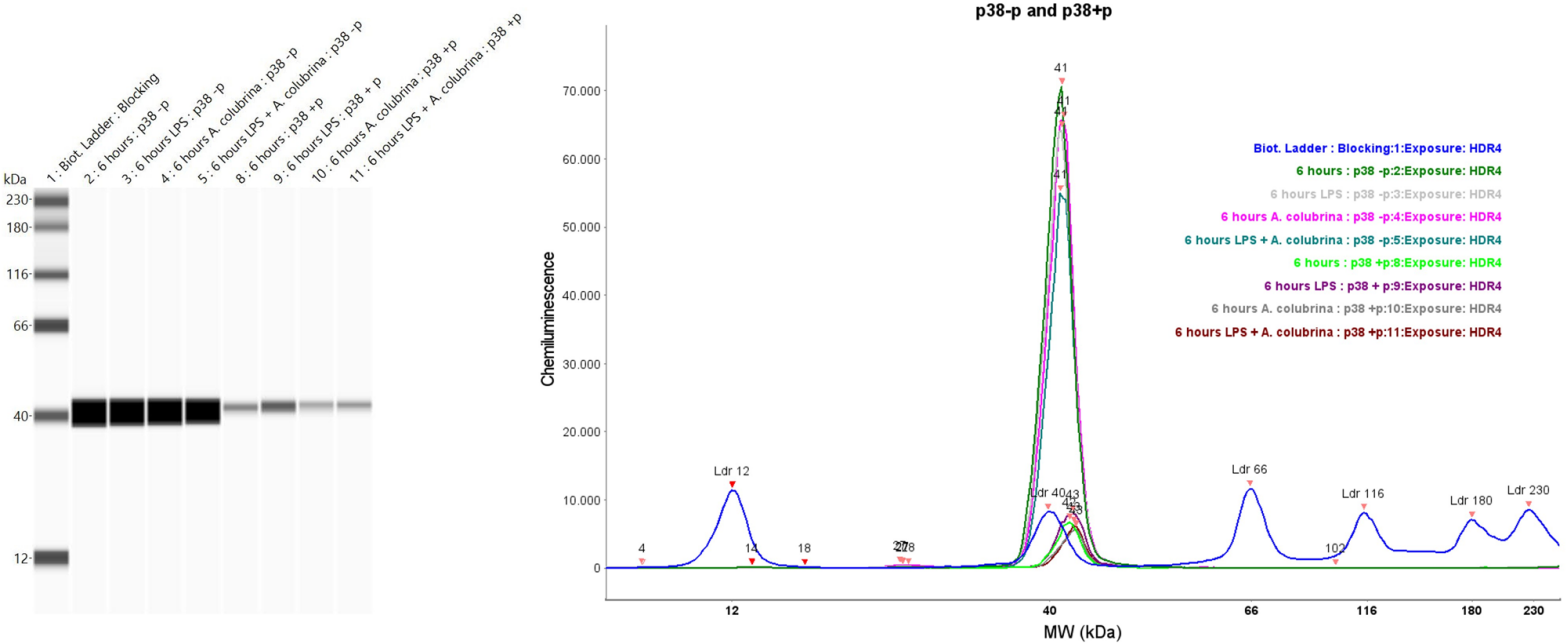
Western blot analysis visualized as virtual blots (left) and peaks (right) of NF-κB + p (phosphorylated form), and p-NF-κB-p (non-phosphorylated form) for THP-1 cells treated for 6 h with *A. colubrina* extract (250 µg/mL) and stimulated by LPS (100 ng/mL).

**Figure 5:**
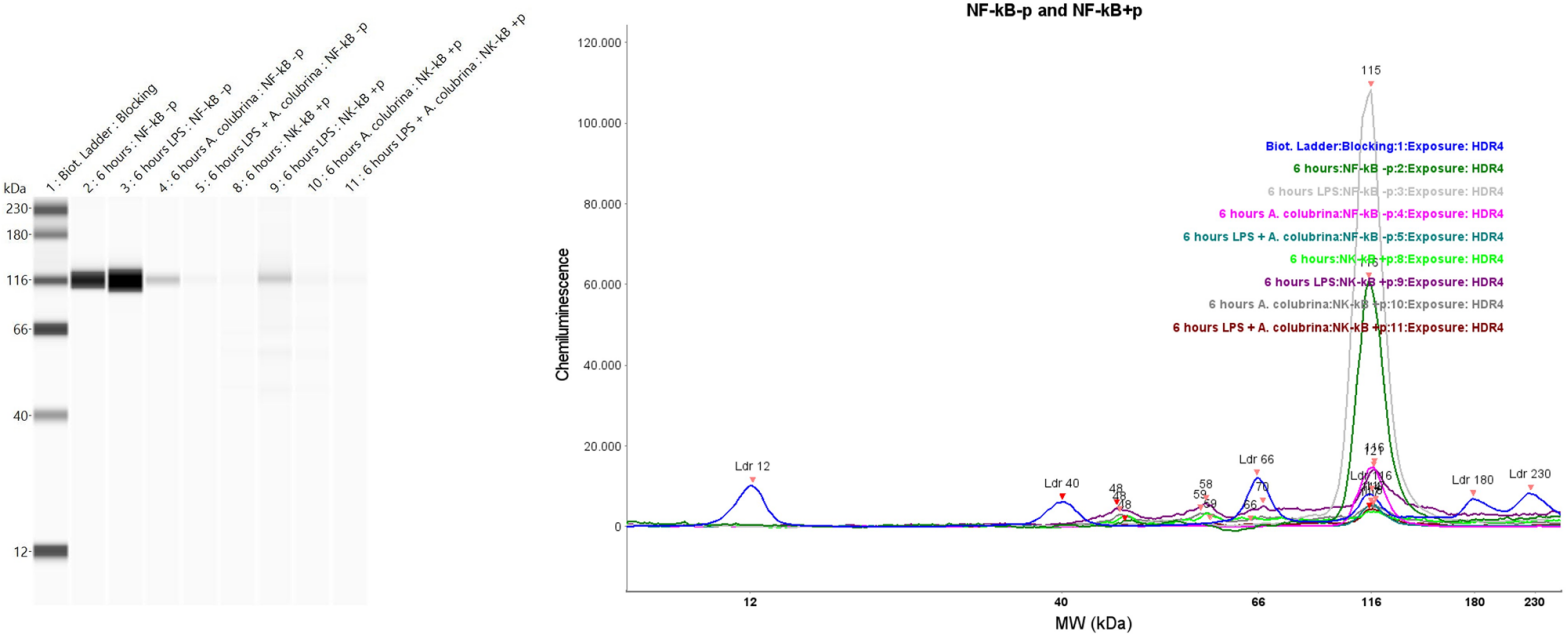
Western blot analysis visualized as virtual blots (left) and peaks (right) of p-38 + p (phosphorylated form) and p38-p (non-phosphorylated form) for THP-1 cells treated for 6 h with *A. colubrina* extract (250 µg/mL) and stimulated by LPS (100 ng/mL).

Therefore, the Simple Western assay data for the regulatory signaling pathways demonstrated that the phosphorylated forms of NF-κB and p38 were detected in all groups tested, with visual differences in bands’ signal intensity. In addition, the LPS-induced cells group triggered NF-κB and p-38 phosphorylated forms. *A. colubrina* also activated the MAPK pathway by itself and in conjunction with LPS through phosphorylation of p38. The same groups also activated the NF-κB (Figs. 4, 5).

### Phytochemical profile

**Figure 6:**
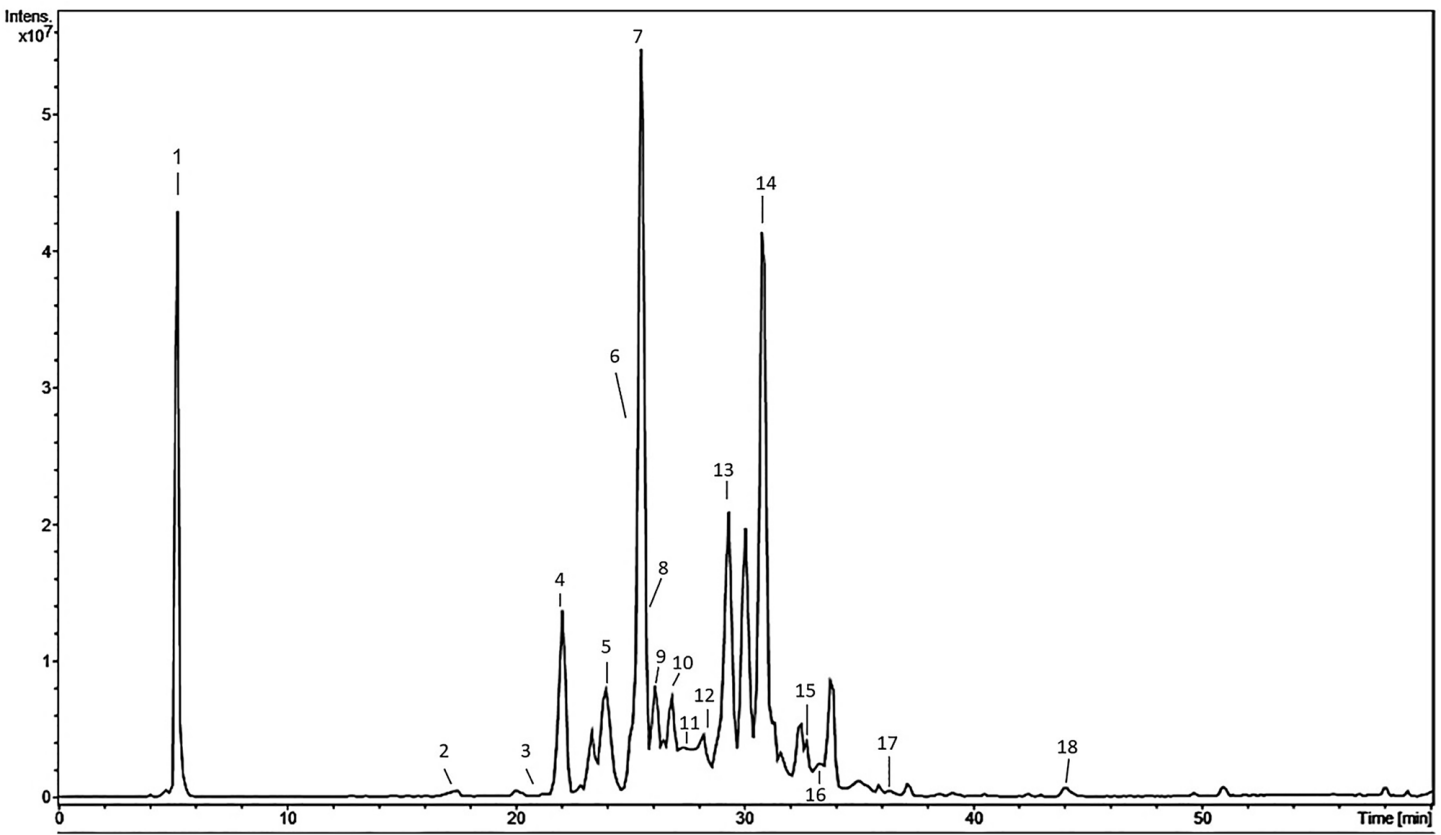
Base peak chromatogram of *A. colubrina* extract.

After the above-cited exciting results, we then performed the phytochemical profile of *A. colubrina* (standardized hydroethanolic extract); it was assessed by HPLC-ESI-MS^n^ (High-Performance Liquid-Chromatography) and HRESIMS (High Resolution Electrospray Ionization Mass Spectrometry), characterized by the presence of flavonoids, predominantly heterosides of catechins, quercetin, kaempferol; and proanthocyanidins (Table 1). The base peak chromatogram is shown in Fig. 6.

**Table 1:** Phytochemical profile of *A. colubrina* extract by HPLC-ESI-MS^n^ and HRESIMS.

Peak	R.T	[M-H]^-^	Formula	Error (ppm)	MS^n^ m/z	Assignment
1	5.3	341.1088	C_12_H_22_O_11_	0.4	MS^2^ [341]: 179; 161; 149; 143; 131 MS^3^ [341 → 179]: 161; 131; 143; 119; 101; 89	Sucrose
2	17.4	451.1244	C_21_H_24_O_11_	0.3	MS^2^ [451]: 289; 245 MS^3^ [451 → 289]: 245; 205; 179	Catechin-O-hexoside
3	21.4	577.1360	C_30_H_26_O_12_	− 1.6	MS^2^ [577]: 425; 407; 289	(E)Catechin-(E)Catechin
4	22.0	561.1401	C_30_H_26_O_11_	0.2	MS^2^ [561]: 451; 409; 391; 289; 271; 245	(E)Fisetinidol–(E)Catechin I
5	23.8	561.1402	C_30_H_26_O_11_	0.1	MS^2^ [561]: 451; 409; 391; 289; 271; 245	(E)Fisetinidol–(E)Catechin II
6	25.2	833.2087	C_45_H_38_O_16_	− 0.0	MS^2^ [833]: 681; 663; 561; 543; 409; 391	B-type Proanthocyanidin Trimer I
7	25.4	561.1405	C_30_H_26_O_11_	− 0.4	MS^2^ [561]: 451; 409; 391; 289; 271; 245	(E)Fisetinidol–(E)Catechin III
8	25.7	545.1448	C_30_H_26_O_10_	1.0	MS^2^ [545]: 527; 435; 409; 289	(E)Guibourtinidol-(E)Catechin I
9	26.1	833.2085	C_45_H_38_O_16_	0.2	MS^2^ [833]: 681; 663; 561; 543; 409; 391	B-type Proanthocyanidin Trimer II
10	26.9	545.1450	C_30_H_26_O_10_	0.6	MS^2^ [545]: 527; 435; 409; 289	(E)Guibourtinidol-(E)Catechin II
11	27.6	713.1506	C_37_H_30_O_15_	0.8	MS^2^ [713]: 561; 409; 391; 289; 271; 245	Galloyl-(E)-afzelechin/fisetinidol -(E)-Catechin
12	28.2	833.2082	C_45_H_38_O_16_	0.6	MS^2^ [833]: 681; 663; 561; 543; 409; 391	B-type Proanthocyanidin Trimer III
13	29.3	833.2089	C_45_H_38_O_16_	− 0.2	MS^2^ [833]: 681; 663; 561; 543; 409; 391	B-type Proanthocyanidin Trimer IV
14	30.9	817.2141	C_45_H_38_O_15_	− 0.4	MS^2^ [817]: 665; 561; 545; 409; 289	(E) -Guibourtinidol—(E) -Afzelechin-(E)-Catechin
15	32.7	801.2179	C_45_H_38_O_14_	1.3	MS^2^ [801]: 765; 545; 409; 289	(E)-Guibourtinidol-(E)-Guibourtinidol-(E)-Catechin
16	33.1	447.0935	C_21_H_20_O_11_	− 0.5	MS^2^ [447]: 285; 255; 163 MS^3^ [447 → 285]: 255; 163	Kaempferol-O-hexoside
17	36.1	463.0891	C_21_H_20_O_12_	− 1.9	MS^2^ [463]: 301 MS^3^[463 → 301]: 271; 255; 179; 151;	Quercetin-O-hexoside
18	44.1	285.0402	C_15_H_10_O_6_	0.9	MS^2^ [285]: 243; 241; 217; 199; 175; 151; 133	Luteolin

The symbol (E) represents both possibilities: catechin or epicatechin; afzelechin or epiafzelechin; fisetinidol or epifisetinidol; guibourtinidol ou epiguibourtinidol. Proanthocyanidin Trimer indicates that there are two possibilities (epi)afzelechin-(epi)afzelechin-(epi)catechin or (epi)fisetinidol- (epi)fisetinidol-(epi)catechin.

## Discussion

The *Anadenanthera colubrina* is a plant that can positively affect human health. Therefore, we studied the standardized hydroethanolic extract and found the *A. colubrina,* in its phytocomplex, potentially interacts with TLRs. Nonetheless, it is yet unknown if its therapeutic effect can be delivered by one specific active compound or by a complex mixture of active substances contained in the extract (the *phytocomplex*). For this reason, identifying bioactive compounds that could act both in modulating the virulence factors of oral microrganism and on the inflammatory response of the host against the pathogen holds promise for improved therapeutic approaches. *A. colubrina* capacity to reduce *C. albicans* infection has been shown in our previous study. However, the approach described was employed for the first time to investigate the immunomodulatory effect of *A. colubrina* extract and its regulation on underlying cell signaling transduction pathways in LPS-induced THP-1 monocytes.

The extract did not affect cell viability at concentrations up to 250 µg/mL, so that it can be a therapeutic application source. Our results are in accordance with several reports that also confirmed the low toxicity *of A. colubrina* on macrophages^22–24^, keratinocytes, and tumoral cell strains^25^.

Several studies have investigated the immunomodulatory effects of medicinal plant extracts using THP-1 cells^26–28^ supporting its application as an in vitro model to study human inflammatory diseases. So, in this study, THP-1 cells were set as the in vitro cell model to evaluate the immunomodulation effects of *A. colubrina*, since these cells possess regulatory proteins which initiate inflammation upon stimulation by LPS^26^. on the regulation of pro-inflammatory cytokine expression during LPS stimulation, through gene expression (transcriptional level) and secretion (proteomic level) of these inflammation mediators.

Considering that TLRs exert pro-inflammatory effects when activated, and anti-inflammatory effects when downregulated or suppressed, they are thought to play a central role in both mediating and modulating inflammatory response^29^. In this regard, we chose a panel of pro-inflammatory markers, since the interaction between TRL4 receptor and LPS induces the activation of NF-κB/MAPK signaling pathways and the release of TNF-α, IL-1β, IL-6 and IL-8 cytokines^12,30,31^. Finally, we evaluated the secretion levels of IL-10 cytokine, as an anti-inflammatory marker.

*A. colubrina* was not effective in reducing the gene expression of IL-8 pro-inflammatory cytokines in the LPS-induced group. However, upon LPS stimuli, *A. colubrina* affected the expression of inflammatory cytokines by down-regulating IL-1β and IL-10 genes expression, which suggests the anti-inflammatory effect of the extract on the transcriptional level of regulation. In addition, our results showed that LPS stimulus effectively up-regulates the gene expression of IL-8 and IL-1β pro-inflammatory cytokines, which proves its effect on inducing transcription of genes related to inflammation responses upon interaction with TRL4 receptor^32^.

Also, we showed that *A. colubrina* itself did not induce any pro-inflammatory and anti-inflammatory response, which is positive considering that the extract did not significantly affect THP-1 cell viability and function. However, upon *A. colubrina* treatment, secretion levels of IL-8 cytokines were not affected in LPS-induced groups, which means that the extract was ineffective in decreasing this pro-inflammatory cytokine in response to the inflammatory stimuli produced by LPS. On the other hand, it is known that IL-1β is vital for the inflammatory host response against pathogens since it is involved in the recruitment of immune cells to the site of infection^33^. Therefore, the study showed a significant decrease in IL-1β secretion by *A. colubrina*, which may prevent additional inflammatory responses induced by the recruitment of immune cells. In addition, IL-10, a representative anti-inflammatory cytokine that plays a critical role in controlling immune responses and is reported to be involved in the inhibition of IL-1β production^34,35^, was significantly enhanced in the present study Therefore, these findings suggest that *A. colubrina* may regulate the immune response by modulating IL-1β and IL-10 cytokines level secretion.

The signaling pathway assay (Simple Western Immunoassay) was used to examine *A. colubrina*-mediated-signal transduction and determine which pathways were affected on the regulation of pro-inflammatory cytokine gene expression, accessing the expression of key proteins involved in NF-κB/MAPK signaling pathways through the detection of phosphorylated forms of NF-κB and p38. MAPK (JNK, ERK, and p38) and NF-κB are crucial intracellular pathways leading to the inflammatory response^36^. These biological responses are mediated by their transcription factors, such as activator protein-(AP1) and NF-κB subunit Iκβα, which are phosphorylated and translocated from the cytoplasm to the nucleus, resulting in an inflammatory action through the expression of target genes, including pro-inflammatory cytokines IL-1β, IL-6, and TNF-α as well as iNOS (inducible nitric oxide synthase) and COX-2 (cyclooxygenase-2) proteins^37,38^.

In this study, we evidenced the boosted activity of phosphorylated NF-κB (NF-κB + *p*) and p38 (p38 + *p*) forms upon LPS stimulation alone in THP-1 cells, which proves the activation from NF-κB/MAPK pathways by LPS, resulting in over-production of inflammatory mediators. In addition, we observed phosphorylation from NF-κB and p38, with an attenuated signal intensity band regarding *A. colubrina* itself and in conjunction with LPS stimuli, especially on the NF-κB transduction factor. These findings suggest that the extract may exert molecular mechanisms involving the modulation of these signaling pathways, particularly on NF-κB protein activation.

Indeed, IL-1β is commonly induced by activating the inflammatory transcription factor NF-κB, which regulates inflammatory responses such as cell proliferation, migration, adhesion, and lymphocyte development^39^. Thus, the decrease in the release of IL-1β cytokine in Luminex analysis in our study is consistent with the downregulation of the NF-κB signaling pathway. Furthermore, considering that the extract combined with LPS was ineffective in decreasing IL-8 levels but significantly decreased IL-1β secretion. Therefore, we hypothesized that phosphorylation of p38, in conjunction with an attenuated activation of NF-κB, was responsible for the interaction between these pathways, determining this final biological response after LPS mediator stimulation^40^.

This approach may also help identify potential therapeutic targets in pathophysiological contexts of exacerbated or chronic inflammation^15,41^. Thus, we reinforce that further analysis to measure the phosphorylation levels of NF-κB and p38 proteins is necessary to verify quantitatively how the extract can regulate the expression of these key proteins related to signaling pathways that trigger the expression and production of pro-inflammatory cytokines.

Besides regulating IL-1β, IL-6, and IL-8, NF-κB/MAPK signaling cascades also monitor the expression of pro-inflammatory cytokine TNF-α^27^. Our study did not evaluate the TNF-α expression. However, recent studies that evaluated the anti-inflammatory effects of *A. colubrina* leaves extract^23^ and protease inhibitors extracted from the same species^24^ showed a significant reduction in TNF-α and nitric oxide (NO) production levels in LPS-induced macrophages. Along with these findings, our results can contribute to understanding the molecular mechanisms underlying the anti-inflammatory effects of *A. colubrina*.

Previously, we evaluated the phytochemical composition of the *A. colubrina* bark extract^25^. It was found a high total polyphenol content (53.18% gallic acid equivalents); tannins (8.77% catechin equivalents) and flavonoids (0.28% quercetin equivalents: mainly heterosides of catechin, quercetin, and kaempferol), and proanthocyanidins. In this current study, we also assessed the chemical profile of the extract by HPLC coupled to Ion Trap and TOF Mass Spectrometry (HPLC-IT-MS/HPLC-TOF-MS). Flavonoids are polyphenolic compounds commonly present in most plants with anti-inflammatory properties^42–44^, supporting the *A. colubrina* immunomodulatory property here evaluated. Additionally, flavonoids can modulate the immune response through the inhibition of molecules that play an essential role in the modulation of mediators related to inflammation response, such as regulatory enzymes and transcription factors, such as NF-κB^10,44–46^.

Several studies have reported that extracts containing flavonoids, such as catechin and glycosylated derivatives of quercetin, can modulate several inflammatory and oxidative stress mediators through the negative regulation of pro-inflammatory cytokines and chemokines (TNF-α, IL-6, IL-1β, IL-8), nitric oxide (NO) and COX-2 in LPS-activated macrophages^42,43,47^. Thus, consistent with these studies, the immunomodulatory effects of *A. colubrina* extract observed in the present study could be related to the presence of flavonoids.

In conclusion, the present in vitro study is the first to show that *A. colubrina* extract has anti-inflammatory properties in LPS-induced THP-1 cells. Furthermore, these effects can be related to modulations in the secretion of IL-1β and IL-10 cytokines through the regulation of intracellular NF-κB and p38-MAPK signaling pathways. Further understanding of additional signaling pathways and activation effects implicated in extract properties might provide novel insights into the immunomodulation and new opportunities for *A. colubrina* rational application. Future analyses are warranted to identify and characterize each substance or group of the substance responsible for the potential anti-inflammatory activity highlighted in the current study; nevertheless, our experimental approach, employing the standardized hydroethanolic extract, is worthy of consideration.

## Methods

### Preparation of the standardized extract

Barks of *Anadenanthera colubrina* (Vell.) Brenan were collected during September in the semi-arid region of Paraíba state, Brazil (7º 22′ 25′′ S, 35º 59′ 32′′ W). It should be noted that all methods used in this study involving plants followed institutional, national, and international guidelines and legislation. Additionally, all necessary permissions and licenses needed for the collection and processing were obtained. Botanical specimens were deposited in the Manuel de Arruda Câmara Herbarium (ACAM) at the State University of Paraíba (UEPB), Campus I, Campina Grande, Paraíba, Brazil, under nº 1936/ACAM. Also, this research was conducted under authorization of Genetic Heritage Management Council, attached to the Brazilian ministry of the environment, SisGen authorization number A289DF4.

The plant material was dried, macerated, and immersed in 80% ethanol for 48 h (10 mg: 25 mL) to obtain a hydroethanolic extract^48^. In addition, the material was filtered three times, vacuum concentrated (Tecnal TE-211, Piracicaba, SP, Brazil), and lyophilized (Martin Christ 1–2 LDplus, Germany), with an extraction yield of 31.7%.

### Cell viability assay

The effect on cell viability of *A. colubrina* extract was evaluated on THP-1 monocyte cells (THP-1 ATCC® TIB-202) and assessed by a resazurin fluorometric method (Cell Titer Blue Viability Assay, Promega Corp, Madison, WI). THP-1 cells were cultured in Roswell Park Memorial Institute medium (RPMI-1640, VWR Life Science, Radnor, PA) supplemented with 10% Fetal Bovine Serum (FBS, Gibco, Invitrogen, Waltham, MA), Penicillin (10,000 U/mL), Streptomycin (10,000 µg/mL), and 2-Mercaptoethanol 50 nM (VWR Life Science, Radnor, PA), at 37 °C in 5% CO_2_. THP-1 cells (2.5 × 10^5^ cells/mL) were seeded in a 24-well plate (Greiner Bio-One North America, Inc Monroe, NC, USA) in RPMI with 10% FBS. The *A. colubrina* extract was diluted in Dimethyl Sulfoxide 1% (DMSO, BDH Solvents, Dawsonville GA), with a final concentration inside the wells of 0.1%, and then added to the cultured cells wells (2500–0.25 µg/mL). After 24 h of incubation at 37 °C in 5% CO_2_, the resazurin (30 µL) was added, and the plates were incubated for 3 h. The fluorescence of the supernatant was read in a microplate reader (SpectraMax M3, Molecular Devices, Sunnyvale, CA), with excitation of 555 nm, emission of 585 nm, and 570 nm *cut-off*^49^. (n = 3 for each group, analyzed on three independent experiments).

### Cell treatment and LPS-induced inflammation assay

As mentioned above, THP-1 cells were seeded in 24-well plates at the same density and culture conditions from the cell viability assay protocol, as mentioned above. Then, the cells were exposed to *A*. *colubrina* extract and LPS from *Porphyromonas gingivalis* (InvivoGen, San Diego, CA), according to the following groups: stimulation with LPS (100 ng/mL), treatment with *A. colubrina* (250 µg/mL), simultaneous exposure to LPS (100 ng/mL) and *A. colubrina* (250 µg/mL), and a control group with no treatment. The plates were incubated for 6 h at 37 °C in 5% CO_2_^50^. Cell supernatants were collected by centrifugation at 1500 rpm for 5 min for quantitative analysis of cytokines. The cells kept on the bottom of the wells were processed for RNA extraction and whole-cell lysate collection for gene expression by RT-PCR and Wes Simple Western assays, respectively.

### Real-time quantitative PCR

RNA was isolated from LPS-induced THP-1 cells and treated with *A. colubrina* using RNeasy® Mini Kit (Qiagen, Hilden, Germany) per the supplier’s specification. In this study were chosen the same host inflammatory cytokine genes that were described in our first report using *A.colubrina*^50^: IL-8 (Qiagen Gene ID#: 3576; Qiagen, Hilden, Germany); IL-10 (Qiagen Gene ID#: 3587; Qiagen, Hilden, Germany); IL-1β (Qiagen Gene ID#: 3553; Qiagen, Hilden, Germany) and GAPDH (*housekeeping*) (Qiagen Gene ID#: 2597; Qiagen, Hilden, Germany). In addition, the gene expressions were normalized to GAPDH. Reactions of qPCR were performed using g QuantiFast® SYBR® Green RT-PCR One Step Kit (Qiagen, Hilden, Germany) in a thermocycler (QuantStudio 3 Real-Time PCR System, Thermo Fischer Scientific, Rockford, IL). The qPCR protocol was as follows: 50 °C for 10 min, 95 °C for 5 min, followed by 40 cycles of 10 s at 95 °C and 30 s at 60 °C. Analysis of relative gene expression was accomplished according to the ΔΔCt method^51^. (n = 3 for each group, analyzed on three independent experiments).

### Luminex for quantitative analysis of inflammatory cytokines

Supernatants from TPH-1 cells treated with *A. colubrina* and exposed to LPS were collected and assayed for analysis expression of pro-inflammatory cytokines IL-8, IL1β, and anti-inflammatory IL-10 using Human Magnetic Premixed Multi-Analyte Luminex Assay Kit (R&D Systems, Minneapolis, MN). Culture supernatants and cytokine capture bead cocktails were incubated overnight. Samples were incubated for 1 h with a biotin-labeled antibody and 30 min with streptavidin-PE. Using Luminex 200 Milliplex System, the data were collected and analyzed using Milliplex Analyst software^52^. (n = 3 for each group, analyzed on three independent experiments).

### WES simple western immunoassay of NF-κB and p38-MAPK

THP-1 cells were lysed with 200 µL of ice-cold Pierce RIPA Lysis Buffer (Thermo Fischer Scientific, Rockford, IL) for 5 min at room temperature to obtain the whole-cell lysate. According to the manufacturer’s instructions, total protein concentration was determined using Pierce BCA Protein Assay Kit (Thermo Scientific, Rockford, IL) to normalize the lysates’ protein content. Wes Simple Western system (ProteinSimple, San Jose, CA) was used to detect the protein expression of NF-κB, p38, phosphorylated NF-κB + p, and phosphorylated p38 + p. According to the manufacturer’s protocol, the Separation Module of 12–230 kDa, Anti-Rabbit Detection Module, and capillary cartridges (ProteinSimple, SM-W002-1, SanJose, CA) were applied. Lysate samples were reduced to 0.4 M dithiothreitol (DTT), mixed with Fluorescent Master Mix, and denatured at 95 °C for 5 min. A biotinylated ladder (12–230 kDa) was used for molecular weight determination. Primary antibodies (1:1000) were used for protein detection of NF-κB and NF-κB -p (MW: 120 kDa), p38 (MW: 40 kDa), and p38 -p (MW:43 kDa) (Cell Signaling Technology, Danvers, MA). The samples, the blocking reagent, the primary antibodies, the HRP-conjugated secondary antibodies, and the chemiluminescent substrate were added to the plate. The WES machine and software (Compass™) provided data as virtual blots (electropherograms) in which the molecular weight and signal intensity are presented^53,54^. (n = 3 for each group, analyzed on three independent experiments).

### Phytochemical profile analysis by LC-ESI-MS^n^ and LC-HRESIMS

The phytochemical profiles of *A. colubrina* extract were analyzed using an HPLC (Shimadzu, Kyoto, Japan) equipped with a C18 column (Kromasil—250 mm × 4.6 mm × 5 μm) coupled to Ion-Trap (Amazon X, Bruker, Berlin, Germany) or microTOF mass spectrometers (Bruker, Berlin, Germany) with an Electrospray Ionization (ESI).First the extract was diluted in methanol (1 mg/mL) and then filtered in a 0.45 µM PVDF (Polyvinylidene Difluoride) membrane. For the chromatography method, methanol (solvent B) and ultrapure milli-Q water were used; the solution was acidified with formic acid (0.1% v/v), following gradient elution of concentration (5 to 100% of solvent B in 60 min). The injection volume was 10 µL, and the flow was set to 0.6 mL/min. Ion Trap and TOF acquisition parameters were as follows: negative ionization mode; spray voltage of 4.4 kV; offset of 500 V; sheath gas at 35 pi; drying gas N_2_; the flow of 8 mL/min and heater temperature of 300 °C. The analysis of the compounds was based on MS/MS data available in the literature.

### Statistical analysis

Data were expressed as the mean ± SEM using one-way analysis of variance (ANOVA) followed by Dunnett’s multiple comparison tests to the vehicle using GraphPad Prism software (version 8.02). (*p* ≤ 0.05 was set as the threshold of significance).

## Data Availability

The datasets used and/or analyzed during the current study available from the corresponding author on reasonable request.
